# Patients with chronic kidney disease and their intent to use electronic personal health records

**DOI:** 10.1186/s40697-015-0058-5

**Published:** 2015-06-09

**Authors:** Tyrone G. Harrison, James Wick, Sofia B. Ahmed, Min Jun, Braden J. Manns, Robert R. Quinn, Marcello Tonelli, Brenda R. Hemmelgarn

**Affiliations:** Department of Medicine, University of Calgary, Calgary, AB Canada; Department of Community Health Sciences, University of Calgary, 3280 Hospital Drive NW, Calgary, T2N 4Z6 AB Canada

**Keywords:** Personal health record, Electronic personal health record, PHR, ePHR, Chronic kidney disease, Patient-centered care

## Abstract

**Background:**

Electronic personal health records (ePHRs) provide patients with access to their personal health information, aiming to inform them about their health, enhance self-management, and improve outcomes. Although they have been associated with improved health outcomes in several chronic diseases, the potential impact of ePHR use in chronic kidney disease (CKD) is unknown.

**Objectives:**

We sought to understand perceptions of CKD patients about ePHRs, and describe characteristics associated with their expressed intent to use an ePHR.

**Design:**

Self-administered paper based survey.

**Setting:**

The study was conducted in Calgary, Alberta, Canada at a multidisciplinary CKD clinic from November 2013 to January 2014.

**Participants:**

Patients with non-dialysis-dependent CKD.

**Measurements:**

Demographics, perceived benefits, and drawbacks of ePHRs were obtained. A univariate analysis was used to assess for an association with the expressed intention to use an ePHR.

**Methods:**

A patient survey was used to determine perceptions of ePHRs, and to identify factors that were associated with intention to use an ePHR.

**Results:**

Overall 63 patients with CKD (76.2 % male, 55.6 % ≥65 years old) completed the survey. The majority (69.8 %) expressed their intent to use an ePHR. CKD patients over the age of 65 were less likely to intend to use an ePHR as compared to those aged <65 years (OR 0.22, 95 % CI: [0.06, 0.78]). Those with post-secondary education (OR 3.31, 95 % CI: [1.06, 10.41]) and Internet access (OR 5.70, 95 % CI: [1.64, 19.81]) were more likely to express their intent to use an ePHR. Perceived benefits of ePHR use included greater involvement in their own care (50.0 % indicated this), better access to lab results (75.8 %), and access to health information (56.5 %). Although 41.9 % reported concerns about privacy of health information, there was no association between these concerns and the intent to use an ePHR.

**Limitations:**

Our results are limited by small study size and single centre location.

**Conclusions:**

We found that patients with CKD expressed their intention to use ePHRs, and perceive benefits such as personal involvement in their health care and better access to lab results. Studies of CKD patients using ePHRs are needed to determine whether ePHR use improves patient outcomes.

## ABRÉGÉ

**Contexte:**

Les fiches électroniques de santé individuelle offrent aux patients l’accès à l’information au sujet de leur santé personnelle, afin de les en informer, de favoriser l’autogestion, et d’améliorer les résultats sur leur santé. Bien que leur utilisation ait été associée à une amélioration des résultats sur la santé dans le cas de plusieurs maladies chroniques, les conséquences possibles de leur utilisation sur l’insuffisance rénale chronique (IRC) demeurent inconnues.

**Objectifs:**

Nous avons cherché à connaître la manière dont les patients atteints d’IRC perçoivent les fiches, de même qu’à décrire les caractéristiques associées à leur intention exprimée d’en utiliser une.

**Type d’étude:**

Un questionnaire en version papier à remplir soi-même.

**Contexte:**

L’étude a été réalisée à Calgary, en Alberta, au Canada, au sein d’une clinique multidisciplinaire de traitement de l’IRC, entre novembre 2013 et janvier 2014.

**Participants:**

Des patients atteints d’IRC, non dépendants de la dialyse.

**Mesures:**

On a obtenu des données démographiques, les avantages perçus, de même que les inconvénients associés à l’utilisation des fiches électroniques de santé individuelle. On a tenté d’établir, au moyen d’une analyse unidimensionnelle, une éventuelle association entre les données et l’intention exprimée par les participants d’utiliser une fiche électronique de santé individuelle.

**Méthodes:**

On a sondé les participants afin de déterminer la manière dont ils perçoivent les fiches électroniques de santé individuelle, et de cibler les facteurs associés à une éventuelle intention d’en utiliser une.

**Résultats:**

Un total de 63 patients atteints d’IRC (76.2 % d’hommes, 55.6 % âgés de ≥ 65 ans) ont rempli le questionnaire. La majorité (69.8 %) a exprimé son intention d’utiliser une fiche électronique de santé individuelle. Les patients atteints d’IRC âgés de plus de 65 ans étaient moins susceptibles d’exprimer une intention d’utiliser une fiche électronique que les patients âgés de moins de 65 ans (RIA = 0.22, IC, 95 % [0.06–0.78]). Ceux qui détiennent une éducation postsecondaire (RIA = 3.31, IC, 95 % [1.06–10.41]) et qui ont accès à Internet (RIA = 5.70, IC, 95 % [1.64–19.81]) étaient plus susceptibles d’exprimer leur intention d’utiliser une fiche électronique de santé individuelle. Les avantages perçus liés à l’utilisation des fiches électroniques comprenaient une participation accrue des patients dans leurs soins (50.0 % l’ont indiqué), un accès amélioré aux résultats de laboratoire (75.8 %), et l’accès aux informations sur la santé (56.5 %). Bien que 41.9 % des patients aient rapporté leurs inquiétudes quant à la confidentialité des informations sur la santé, il n’y avait aucune association entre ces inquiétudes et l’intention des patients d’utiliser une fiche électronique de santé individuelle.

**Limites de l’étude:**

Nos résultats sont limités en raison de l’étendue restreinte de l’étude et du fait que celle-ci ne couvre qu’un seul centre.

**Conclusions:**

Nous avons trouvé que les patients atteints d’IRC avaient exprimé leur intention d’utiliser les fiches électroniques de santé individuelle, de même que les avantages perçus tels que la participation individuelle à leurs propres soins de santé et un meilleur accès aux résultats de laboratoire. Des études sur les patients atteints d’IRC qui utilisent les fiches électroniques de santé individuelle sont nécessaires afin de déterminer si l’utilisation de ces fiches améliore les résultats sur leur santé.

## What was known before

The use of ePHRs is associated with improved health outcomes in many chronic diseases.

## What this adds

CKD patients are interested in the use of ePHRs in their care. Many benefits are perceived, and although 41.9 % were concerned about health privacy, this was not associated with expressed intention (or lack of intention) to use an ePHR. Although those aged 65 or older were not as likely to express interest in an ePHR as their younger counterparts, this allows for targeted implementation of these technologies among those that may not use it otherwise.

## Background

There is increasing emphasis on the use of health-related technology to improve care and increase patient self-management of their conditions [1–3]. Electronic health records are increasingly common and allow providers to record visit history, test results, medications, and treatment plans, among other functions [3, 4]. There has been interest in allowing patient access to these electronic records, and the creation of electronic personal health records (ePHRs) to keep patients updated on the status of their health conditions and facilitate self-management of their medical conditions [5, 6]. ePHRs offer more to patients than simply viewing test results; they allow for increased patient involvement in their own medical decision-making in concordance with the goal of patient-centred care. This is in part related to widespread Internet use and ease of access that patients have to medical information in general [5, 7]. Recent studies suggest that patients who regularly use the Internet are more than three times more likely to search for health-related information on the Internet than from health care providers [8]. This suggests a potentially important role for ePHRs.

Despite the increasing prevalence of older adults with chronic disease, and the emphasis on patient self-management [9], little research has been conducted regarding use of ePHRs. There are studies on ePHR use in diabetic populations, and among patients with prostate cancer and congestive heart failure, but information on factors associated with use of the ePHR is limited. Among patients with diabetes, ePHR use was associated with improvements in process of care measures including blood pressure and hemoglobin A1C [10]. Use of ePHRs in patients with congestive heart failure [11] and prostate cancer [12] demonstrated benefit including improving user satisfaction, access to lab results, and increased communication with health care providers. Information on ePHR use in CKD populations is also limited, particularly in North America [6]. Electronic health records utilized by physicians treating CKD have been shown to improve rates of AVF placement prior to starting dialysis, and initiate dialysis more often as an outpatient rather than emergent initiation in hospital [13, 14]. Although these physician tools have been shown to be useful, patient-utilized ePHRs have been recently identified as a new tool for use in CKD management as well [3, 6]. A cohort of 11,352 patients with CKD in the United Kingdom was studied recently after having access to an ePHR for several years [15]. They were able to show that almost three quarters of patients that initially signed up persisted in using the ePHR for a median of 18.9 months. This level of patient uptake is almost double what has been described in other chronic diseases such as diabetes and hypertension [16, 17].

Patients with chronic kidney disease (CKD) may benefit from ePHRs due to their regular follow-up appointments, frequent tests, and need for education on disease and lifestyle topics [18]. However to effectively implement ePHRs, patient perceptions are important to consider [19]. We sought to investigate how ePHRs are perceived by patients with CKD, and to examine factors that are associated with their intent to use ePHRs.

## Methods

### Study and questionnaire design

Non-dialysis dependent CKD patients attending a multidisciplinary CKD Clinic in Calgary, Alberta, Canada were invited to complete a questionnaire regarding their intended use and access to ePHRs. The survey was pilot-tested for face validity and clarity with 5 CKD patients and 3 nephrologists, and modified based on their feedback. The survey included data on patient demographics (age, gender, education, self-rated health), details of personal health management (interest in maintaining records, method of record maintenance, access to internet/online tools, perceptions on the availability and adequacy of personal health information), and perceptions of ePHR use (potential benefits and drawbacks). In addition, patients were asked if they intended to use an ePHR if it were to become available through the clinic on a 5-point Likert scale from “strongly disagree” to “strongly agree.” Ethics approval was granted by the Conjoint Health Research Ethics Board at the University of Calgary.

### Setting and participant selection

We invited English-speaking and literate patients at the outpatient multidisciplinary CKD clinic in Calgary to participate in the paper-based survey over a three-month period (Nov 2013–Jan 2014). The clinic includes nephrologists, nurse clinicians, pharmacists, dieticians, and social workers that use a case management approach to care for patients with CKD [20]. Eligible patients were non-dialysis dependent, with estimated glomerular filtration rates (eGFRs) of less than 60 mL/min/1.73 m^2^. We did not collect data on patients who refused to complete the survey.

### Data analysis

Descriptive statistics were used to summarize patient characteristics and perceived benefits and concerns with ePHR use. Intention to use the ePHR was determined by responding “agree” or “strongly agree” when asked if they intend to use the ePHR if it became available. Univariate analysis was undertaken to determine the association between each variable and participants’ expressed intent to use an ePHR. We were unable to undertake multivariable analysis due to the study size. Analyses were conducted using STATA, version 11.2 [21].

## Results

### Descriptive results

A total of 63 patients completed the survey. Characteristics of participants are presented in Table 1, stratified by their expressed intent to use an ePHR. Overall, the majority of participants were 65 years of age or older (55.6 %), male (76.2 %), and had at least some post-secondary education (51.6 %). Over half (52.4 %) rated their health as fair or poor. The majority of participants (76.2 %) reported regular use of the Internet, and believed patients should have access to their own medical information (75.8 %). Importantly, 69.8 % of our patient group intended to use an ePHR if it became available.

**Table 1:** Baseline characteristics; overall and by expressed intent to use the ePHR^a^

Characteristic	Overall	Intend to use ePHR *n* = 44	Don’t intend to use ePHR *n* = 19
	N (%)	N (%)	N (%)
Age			
<65 Years	28 (44.4)	24 (54.5)	4 (21.1)
≥65 Years	35 (55.6)	20 (45.5)	15 (78.9)
Gender			
Male	48 (76.2)	34 (77.3)	14 (73.7)
Female	15 (23.8)	10 (22.7)	5 (26.3)
Education			
No Post-secondary	30 (48.4)	17 (39.5)	13 (68.4)
Post-secondary	32 (51.6)	26 (60.5)	6 (31.6)
Self-Perceived Health			
Fair or Poor	33 (52.4)	26 (59.1)	7 (36.8)
Good to Excellent	30 (47.6)	18 (40.9)	12 (63.2)
Current use of Internet			
No	15 (23.8)	6 (13.6)	9 (47.4)
Yes	48 (76.2)	38 (86.4)	10 (52.6)
Believe patients should have access to personal medical information			
No	15 (24.2)	5 (11.4)	10 (52.6)
Yes	47 (75.8)	39 (88.6)	8 (42.1)

^a^Some participants did not respond to all questions. Percentages were calculated based on the number of respondents for each question

Among participants that expressed their intent to use the ePHR if made available, the majority were younger than 65 years of age (54.5 %), with an equal distribution of males and females (Table 1). The majority of patients who expressed interest in ePHR use had post-secondary education (60.5 %), perceived their health as fair or poor (59.1 %), currently used the Internet (86.4 %), and believed that patients should have access to personal medical information (88.6 %).

Among participants that indicated they did not intend to use an ePHR, the majority were age 65 and older (78.9 %) (Table 1). Most did not have post-secondary education (68.4 %), and half currently used the Internet (52.6 %). Only 42.1 % believed that patients should have access to their medical information.

### Perceived benefits and drawbacks of ePHR Use

**Fig. 1:**
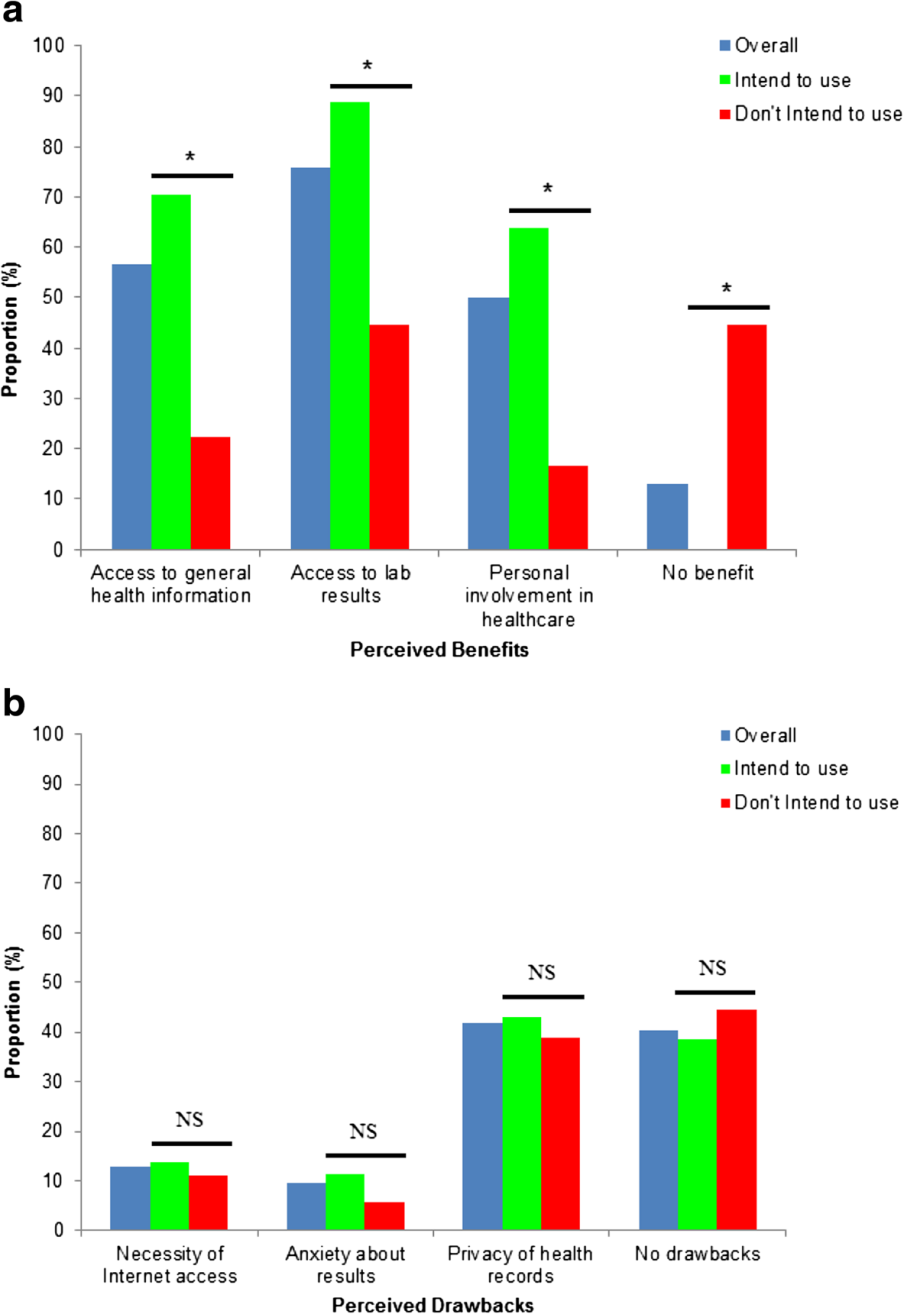
**a** Perceived benefits of ePHR use stratified by expressed intent to use ePHRs. * Indicates significant difference in indication of perceived benefit between those who ‘intend to use’ and those who ‘don’t intend to use’ (*p* < 0.05). **b** Perceived drawbacks of ePHR use stratified by expressed intent to use ePHRs. NS indicates non-significance between those who ‘intend to use’ and those who ‘don’t intend to use’

Patients were asked about potential benefits and drawbacks of ePHR use. Overall, 56.5 % of patients reported the benefit of having access to general health information (Fig. 1a). When separated based on intent to use the ePHR, 70.5 % of those who intended to use an ePHR thought this was a benefit, compared with 22.2 % of those who did not express intent to use the ePHR. Similarly, 84.4 % of those who intended to use an ePHR thought access to lab results was a key benefit, while 75.8 % of the cohort as a whole felt this was a key benefit. Half (50.0 %) of the total group felt that more personal involvement was a benefit, and this number increased to and 63.6 % among those who intended to use the ePHR. Patients who did not convey intent to use the ePHR did not report anticipated benefit of ePHR use as often, with 44.4 % reporting no anticipated benefit.

With respect to potential drawbacks of ePHR use, lack of Internet access or use was not perceived to be a major drawback (12.9 %), nor was anxiety about results (9.7 %) (Fig. 1b). Privacy of health records was the most common concern noted regarding ePHR use in our survey population, with 41.9 % reporting this as a potential concern.

### Factors associated with expressed intention to use an ePHR

In a univariate analysis, older age (≥65 years) was associated with a lower likelihood of expressed intent to use an ePHR (OR 0.22, 95 % CI: [0.06, 0.78]), while there was no association between gender or self-perceived health and intent to use the ePHR (Table 2). Patients with post-secondary education were more than three times as likely to indicate they intended to use the ePHR compared to those with lower levels of education (OR 3.31, 95 % CI: [1.06, 10.41]). Internet access was also significantly associated with greater expressed intent to use an ePHR (OR 5.7, 95 % CI: [1.64, 19.81]).

**Table 2:** Univariate odds ratios for the association of demographics and perceptions of ePHRs with expressed intent to use the ePHR

Characteristic	OR	95 % Confidence interval
Demographics		
Age		
<65 years	Reference	
≥65 years	0.22*	(0.06, 0.78)
Education		
No post-secondary	Reference	
Post-secondary	3.31*	(1.06, 10.41)
Self Perceived Health		
Fair/Poor	Reference	
Good to Excellent	0.40	(0.13, 1.22)
Gender		
Male	Reference	
Female	0.82	(0.24, 2.85)
Has Internet Access		
No	Reference	
Yes	5.7**	(1.64, 19.81)
Benefits		
More personal involvement		
No	Reference	
Yes	8.35**	(2.31, 30.20)
Access to general health information		
No	Reference	
Yes	9.75**	(2.62, 36.34)
Access to lab results		
No	Reference	
Yes	8.75**	(2.19, 34.90)
Drawbacks		
Necessity of Internet Access		
No	Reference	
Yes	1.26	(0.23, 6.94)
Privacy of records		
No	Reference	
Yes	2.18	(0.24, 20.09)
Anxiety about results		
No	Reference	
Yes	1.19	(0.39, 3.66)

*p < 0.05; **p < 0.01

The perceived benefit of greater personal involvement in healthcare was associated with expressed intent to use (OR 8.35, 95 % CI: [2.31, 30.20]), as was the benefit of access to health information (OR 9.75, 95 % CI: [2.62, 36.34]). Likewise, the perceived benefit of access to lab results was associated with intention to use the ePHR (OR 8.75, 95 % CI: [2.19, 34.90]). With respect to drawbacks of the ePHR, concern over the necessity of Internet use, and anxiety over test results were not associated with participants indicating they intended to use the ePHR. Despite the prevalent concern over health record privacy, there was no association between this concern and expressed intent or lack of intent to use ePHRs.

## Discussion

In our survey of patients with CKD managed in a multidisciplinary clinic, we found that the majority of respondents felt that patients should have access to their personal health information, and almost three-quarters indicated that they would use an ePHR if it were available.

Our results suggest that CKD patients who are younger, have post-secondary education, and have access to the Internet are more likely to express interest in using an ePHR, while gender and perceived health status were not associated with expressed intent to use an ePHR. Our results regarding age are consistent with results of more than ten thousand patients looking at the association between ePHR use and diabetic control, with younger patients being more likely to use an ePHR [10]. It is well known that older adults are selective in their uptake of modern information technologies [22, 23], which has important implications for implementation of ePHRs in the CKD population, in particular. In a UK study that looked at ePHR uptake in CKD patients, they found that the younger cohorts of patients had greater persistent ePHR use over the observed four years of use when compared to the patients greater than 75 years of age, findings which are consistent with ours [15]. There is some evidence, however, that identifies the elderly as being more capable of using health-related technology than ever before. Recently, a group of Taiwanese patients with prostate cancer was given a quality-of-life questionnaire both in paper and electronic form [24]. Although almost 80 % had no prior computer use, 87.0 % of patients over 70 years old felt the electronic survey was easy to use, and 59.2 % preferred the electronic version. This trend persisted even among those who had never used a computer before. Despite a proportion of our CKD patients being elderly and not having Internet access or using computers, they still may be able to successfully utilize the ePHR as evidenced by this study. In addition, the data suggest that patients with higher levels of education were more likely to intend to use ePHRs, which is similar to the aforementioned diabetic population with ePHR access. This information may prove important for the targeted introduction of ePHRs in the future in CKD. In particular, our study has identified an important subgroup of elderly, less-educated patients without Internet access, whereby resources may be directed to ensure they are educated and able to utilize the ePHR.

Although not statistically significant, we found that patients with a lower perceived health status intended to use the ePHR more often. This is similar to patients with congestive heart failure, where those with worse symptoms were more likely to use an ePHR [11]. We also found that patients that identified the benefits of greater personal involvement in their healthcare, greater access to health information and lab results had greater expressed intent to use ePHRs. A Canadian consumer survey of ePHR perceptions found that perceived usefulness of the ePHR was the single most important factor for intention to use [25]. Similar results were also reported in a study of US military personnel [4]. These studies support the proposition that patients who perceive benefits of the ePHR will use it when it becomes available. As most of our patients identified numerous benefits to an ePHR, perhaps this will translate to ePHR use as well.

Although a large proportion of our participants (41.9 %) identified the concern of health information privacy, this was not associated with patient’s expressed intent or lack of intent to use ePHRs. The level of concern over privacy reported in the literature varies. Only 6 % of a Canadian prostate cancer cohort reported that they were concerned with privacy after implementation of the ePHR [12]. Among a cohort of 3874 military veteran patients using an ePHR, 32.9 % reported concern over privacy at baseline, which increased to 36.6 % after a year of ePHR use (*p* < 0.001) [26]. Although the concerns over privacy were shown to significantly increase after ePHR implementation, the effect on ePHR utilization rates or patient outcomes is unclear.

The results of our study should be interpreted in light of its limitations. Our study was limited by a small sample size from a single centre, which may have contributed to the lack of statistical significance for some of our variables. Sample size also limited the ability to complete a multivariate analysis of variables associated with expressed intent to use. Larger studies are needed to investigate this further. Currently, large CKD registries are being developed in North America with the intent of analyzing the benefit and impact of health care provider electronic health records and ePHRs [3]. Although our study focused on patients in a single CKD clinic, the patient demographics are similar to CKD patients in much larger cohort studies in North America [27]. Importantly, the difference between expressed intent to use the ePHR and actual adoption of the ePHR has yet to be investigated.

## Conclusions

Our findings suggest that patients with CKD were interested in accessing their personal health information and expressed intent to use ePHRs, if available. Perceived benefits of ePHR use included greater involvement in personal health care and better access to health information and lab results. Factors such as security, anxiety about their results, and lack of Internet access did not affect their expressed intent to use ePHRs. Further research is needed to determine whether intent will correlate with actual ePHR use, and whether ePHRs improve patient outcomes in CKD.

## Abbreviations

CKD: Chronic kidney disease
eGFR: Estimated glomerular filtration rate
PHR: Personal health record
ePHR: Electronic personal health record
